# Experiences of Care and Gaslighting in Patients With Vulvovaginal Disorders

**DOI:** 10.1001/jamanetworkopen.2025.9486

**Published:** 2025-05-08

**Authors:** Chailee F. Moss, Arthi Chinna-Meyyappan, Gabriela Skovronsky, Jessica Holloway, Sylvia Lorenzini, Na’imah Muhammad, Isabella Kopits, Sara Perelmuter, Leia Mitchell, Mollie Rief, Jill Krapf, Caroline Pukall, Andrew Goldstein

**Affiliations:** 1 The Centers for Vulvovaginal Disorders, Washington, DC; 2 Department of Psychology, Queen’s University, Kingston, Ontario, Canada; 3 Weill Cornell School of Medicine, New York, New York

## Abstract

**Importance:**

Medical gaslighting, in which a patient’s concerns are dismissed without proper evaluation, has been described anecdotally in vulvovaginal patient care, but has not been quantified.

**Objective:**

To use a patient-centered instrument to measure adverse experiences in vulvovaginal care.

**Design, Setting, and Participants:**

Common themes from National Vulvodynia Association patient testimonials were used to design a mixed-methods measure of patient experience that included both quantitative and qualitative questions. An instrument was created and submitted to officers from the National Vulvodynia Association and Tight-Lipped, another patient advocacy organization, for feedback. The measure was then completed by patients before their first appointment at a vulvovaginal disorder referral clinic from August 2023 to February 2024.

**Exposure:**

Participation in the survey.

**Main Outcomes and Measures:**

The primary outcome was the incidence of reported clinician behavior and consequent distress as reported on the survey instrument. Quantitative data were analyzed using simple descriptive statistics (mean [SD], median [IQR], and percentage). Narrative responses provided by patients were analyzed using the clinical-qualitative method for content analysis.

**Results:**

A total of 520 patients completed surveys; 5 were eliminated because the patient was younger than 18 years, 6 were eliminated for duplication, 6 were eliminated because they had no past clinician, and 56 were eliminated for completely blank responses. Thus, surveys of 447 patients (mean [SD] age, 41.7 [15.2] years) were analyzed (86% response rate). Patients had a mean (SD) of 5.50 (4.53) past clinicians. Patients reported that a mean (SD) of 43.5% (33.9%) of past practitioners were supportive, 26.6% (31.7%) were belittling, and 20.5% (30.9%) did not believe the patient. In total, 186 patients (41.6%) were told they just needed to relax more, 92 (20.6%) were recommended to drink alcohol, 236 (52.8%) considered ceasing care because their concerns were not addressed, 92 (20.6%) were referred to psychiatry without medical treatment, 72 (16.8%) felt unsafe during a medical encounter, and 176 (39.4%) said they were made to feel crazy, the most distressing surveyed behavior (rated at a mean [SD] of 7.39 [3.06] of 10 on a numerical rating scale of distress). A total of 1150 quotations were analyzed qualitatively; common themes included lack of clinician knowledge (247 quotations) and dismissive behaviors (211 quotations).

**Conclusions and Relevance:**

In this cross-sectional study, a patient-centered measure of adverse experiences in vulvovaginal care was developed. Participants reported common past experiences with gaslighting and substantial distress; they frequently considered ceasing care. There is an urgent need for education supporting a biopsychosocial, trauma-informed approach to vulvovaginal pain and continued development of validated instruments to quantify patient experiences.

## Key Points

**Question:**

How can patient experiences in vulvovaginal disorders be quantified, and what are the results of such quantification?

**Findings:**

This cross-sectional study used a survey developed with patient advocacy organizations that garnered 447 patient responses; 52.8% of patients considered stopping care because of their experiences. Patient narratives noted substantial clinician knowledge and behavioral deficits.

**Meaning:**

These findings suggest that patients with vulvovaginal disorders experience harm from knowledge gaps and certain clinician behaviors; educational and behavioral interventions have substantial potential to improve patient care.

## Teaser

This cross-sectional study uses a patient-centered survey instrument to measure patients’ adverse experiences in vulvovaginal care.

## Introduction

Epistemic injustice—injustice related to knowledge and knowing—is an important consideration in relationships affected by a power differential.^1^ Epistemic injustice can manifest as unfair dismissal of a person’s knowledge of their own experience; it may also comprise unfairly preventing a person from obtaining or sharing knowledge about their experience.^1^ *Gaslighting* is a specific form of epistemic injustice in which dismissal, usually by a figure in power, causes the recipient of the dismissal to question their own perception of reality.^1,2^ Gaslighting has been described as it relates to politics, interpersonal relationships, employment practices, social work, and many other settings.^2,3,4^ *Medical gaslighting* has been defined as, “…an act that invalidates a patient’s genuine clinical concern without proper medical evaluation, because of physician ignorance, implicit bias, or medical paternalism.”^5^ In medicine, the clinician-patient relationship is certainly vulnerable to gaslighting, in which disbelief in patient report (testimonial injustice) may cause a patient to question their own experience of illness.^4,6^

Epistemic injustice has been examined in several medical contexts, including chronic fatigue syndrome, fibromyalgia, response to intimate partner sexual violence, and long COVID-19.^7,8,9,10^ In reproductive health, such injustices have been described in obstetrics, where lack of testimonial justice affects maternal trust, morbidity, and mortality.^11^ Gynecology patients, and in particular those experiencing pain, are subject to numerous discriminatory phenomena at the structural and individual level. These factors lead to insufficient treatment of pain, delays in diagnosis, and pronounced psychological distress, among a myriad of other harms.^12,13,14,15^

Peer-reviewed sources about specific gynecologic contexts are limited; one analysis of transgender and nonbinary people’s perception of endometriosis care detailed the harmful effects of dismissive and gaslighting behavior.^16^ Publicly available doctoral theses have also explored the substantial effect gaslighting can have on traumatic stress in patients from minoritized racial and ethnic groups with endometriosis, as well as the general effect of symptom invalidation in this condition.^17,18^ In the reproductive endocrinology literature, it has been noted that differences in interactions with health care clinicians affect racial disparity in infertility care, but few studies have directly addressed epistemic injustice in this context.^19^

The association of epistemic injustice with patients’ perception of care or willingness to seek care has not been described in the unique context of vulvovaginal pain, nor do patient-centered instruments exist to characterize such associations. The present study aimed to develop a patient-centered measure to assess experiences of a spectrum of types of epistemic injustice in patients seeking care for vulvovaginal disorders. The study also aimed to describe patient experiences by administering the instrument to patients establishing care at a referral center for vulvovaginal disorders and analyzing both their quantitative and qualitative (narrative) responses.

## Methods

The survey used in this cross-sectional study was approved by the BRANY institutional review board as exempt from having to obtain informed consent under categories 2(iii) and 4(iii), as detailed in 45 CFR 46.104(d), and BRANY’s standard operating procedures. Reporting was undertaken in keeping with Strengthening the Reporting of Observational Studies in Epidemiology (STROBE) reporting guidelines for cross-sectional studies.

### Instrument Design

Testimonials of patients available on the National Vulvodynia Association (NVA) website were evaluated by investigators (C.F.M., J.H., S.L., G.S., N.M., and I.K.), and common themes were extracted and used to design a survey instrument. Twenty-three narrative testimonials are available on the website and were submitted voluntarily by patients (these do not include demographic or clinical information).^20^ The mixed-method instrument, Gaslighting And Sexual Medicine, included 37 items and was administered via Survey Monkey (eAppendix in Supplement 1). The questionnaire comprised both quantitative questions for statistical analysis and open-text fields to elicit narrative responses. Survey items were designed to quantify both the incidence of reported clinician behavior and consequent distress. Responses included multiple choice numerical options, binary yes or no fields, and 12 items composed of 0 to 10 numerical scales to allow patients to quantify the magnitude of distress caused by clinician behaviors surveyed in yes or no questions. Each survey item was followed by an open-text field so patients could elaborate in any manner they chose beyond their numerical response. The survey instrument was subsequently submitted to officers from 2 organizations that advocate for patients with vulvovaginal disorders: the National Vulvodynia Association and Tight-Lipped. Feedback from organization officers was then used to modify the survey. On the basis of this feedback, items 6, 18, 19, and 35 were added to the questionnaire.

### Survey Administration

The survey was offered to patients establishing care at a center specializing in vulvovaginal disorders, prior to initial consultation from August 2023 to February 2024. The optional survey was collected using SurveyMonkey. In addition to the questionnaire, some sociodemographic information was collected. Age and self-reported race and ethnicity were collected because gaslighting and other harmful clinical behaviors may disproportionately affect groups that are marginalized on the basis of their social locations. Patients were given options to select responses and to provide content in open-text fields if they felt the categories were not applicable to them or if further explanation was needed. In addition, responses to validated instruments, including the Female Sexual Function Index, the revised Female Sexual Distress Scale, the Pain Catastrophizing Scale, the short form of the Pain Anxiety Symptom Scale, the depression screening instrument Patient Health Questionnaire–9, and General Anxiety Disorder instrument, were collected.^21,22,23,24,25,26,27^ The sample size was based on the number of patients establishing care in the allotted time period.

### Statistical Analysis

#### Quantitative Data Analysis

Patients were eliminated who were younger than 18 years, did not complete any survey questions, or reported that they had not seen any clinician for their concern prior to establishing care with the clinic. Data were analyzed using simple descriptive statistics (mean [SD], median [IQR], and percentages) in Excel (Microsoft). SPSS statistical software version 29.0.2.0(20) (IBM) was used to calculate Spearman rank correlation coefficients. Statistical significance was set at 2-sided *P* < .05.

#### Qualitative Data Analysis

Narrative responses provided by patients were analyzed using the clinical-qualitative method for content analysis. The researchers (C.F.M., J.H., and S.L.) independently and cooperatively completed the following steps: editing material for analysis, floating reading, construction of codes of meaning, general refining of the codes and the construction of categories, discussion, and validity.^13^ Full-text responses were tabulated in a spreadsheet and authors independently reviewed responses. Responses with similar meaning were grouped and impactful responses were highlighted, focusing on what the patient expressed in each passage. The units of analysis were given codes of meaning, and the full text was again reviewed to gather material for analysis under each code. Each participant’s response was reviewed to refine and expand the codes. The collated data were then categorized in relation to the research question, ensuring categories were exhaustive and mutually exclusive. Representative and rich quotations were extracted for each category and then reviewed by authors to ensure validity.

## Results

Of 520 administered surveys, surveys were eliminated for age younger than 18 years (5 surveys), duplication (6 surveys), no past clinicians (6 surveys), and lack of any responses (56 surveys). In total, 447 patients (86%) completed the survey in part or in full (mean [SD] age, 41.7 [15.2] years; range, 18.6-83.5 years). Patients’ self-reported race and ethnicity are shown in Table 1.

**Table 1.** Demographic Characteristics of Participants

Characteristic	Participants, No. (%)
Race	
African American or Black	13 (2.9)
American Indian	4 (0.9)
Asian	14 (3.1)
White	397 (90.4)
Other^a^	26 (5.8)
Ethnicity	
Spanish, Hispanic, or Latinx descent	55 (12.3)
Cuban	3 (0.7)
Mexican	10 (2.2)
Puerto Rican	8 (1.8)
Other^b^	19 (4.3)
Age, mean (SD), y	41.7 (15.2)
Instrument score, mean (SD)	
Female Sexual Function Index^c^	15.9 (10.5)
Female Sexual Distress Scale Revised^d^	29.1 (13.6)
Patient Health Questionnaire–9^e^	5.59 (5.46)
Pain Anxiety Symptom Scale^f^	31.3 (21.4)
Pain Catastrophizing Scale^g^	20.2 (14.2)

^a^ Includes the following patient-described categories: Ashkenazi Jewish, Eastern European, Hispanic, Hispanic Mix, Hispanic/Latino, Indian, Italian, Jewish, Latino, Middle Eastern, and White British.

^b^ Includes the following patient-described categories: 90% Spanish, 10% rest of the world; Brazilian-Portuguese; Colombian; Ecuadorian/Argentinian; Guatemalan; half Argentine; half Spanish half German; and Peruvian.

^c^ This index is scaled out of 30 total points.

^d^ This is a 52-point scale, and a score greater than 11 indicates female sexual distress.

^e^ This is a 27-point depression screening instrument, with a score of 5 to 9 indicating mild depression, 10 to 14 indicating moderate depression, 15 to 19 indicating moderately severe depression, and 20 to 27 indicating severe depression.

^f^ Range 0 to 100, with higher score indicating more pain-related anxiety.

^g^ Range 0 to 52, with score greater than 30 considered statistically significant.

Patients had a mean (SD) of 5.50 (4.53) past clinicians. Survey responses for questions related to the proportion of past clinicians engaging in supportive or gaslighting behavior can be found in Table 2. Patients reported that a mean (SD) of 43.5% (33.9%) of past clinicians were supportive, 26.6% (31.7%) were belittling, and 20.5% (30.9%) did not believe the patient. Patient responses to binary questions about past experiences of specific clinician behaviors can be found in Table 3, along with the distress ratings experienced based on these behaviors. A majority of patients (236 patients [52.8%]) were told their physical examination findings were normal despite substantial pain during their examination. In total, 186 patients (41.6%) were told they just needed to relax more, 92 (20.6%) were recommended to drink alcohol, 92 (20.6%) were referred to psychiatry without medical treatment, 72 (16.8%) felt unsafe during a medical encounter, and 176 (39.4%) said they were made to feel crazy. Patients were most distressed by a common definition of gaslighting (being made to feel crazy) and reported a mean (SD) distress score of 7.39 (3.06) of 10 for this question. Most patients considered stopping care, either because their concerns were not being addressed (236 patients [52.8%]) or because they did not feel there were other practitioners who could help them (254 patients [56.8%]).

**Table 2.** Quantitative Patient Report of Past Clinician Behaviors

Question	Past clinicians reported			Association with patient numerical rating of overall distress		Association with considering giving up seeking care	
	Total No., mean (SD)	Percentage, mean (SD)^a^	Percentage, median (IQR)	Spearman ρ	*P* value	Spearman ρ	*P* value
How many medically trained health care providers have you seen to address your pelvic/vulvar pain in the past?	5.50 (4.53)	NA	NA	0.479	<.001	0.291	<.001
Of the health care providers you have seen, how many have partnered with you in making the plan for your care?	1.85 (1.55)	43.5 (33.9)	36.0 (16.6-66.7)	−0.391	<.001	−0.285	<.001
Of the health care providers you have seen, how many made you feel supported?	1.82 (1.50)	43.6 (35.3)	33.3 (20.0-60.0)	−0.476	<.001	−0.324	<.001
Of the health care providers you have seen, how many made you feel like they were not really listening to your symptoms?	2.85 (3.59)	47.5 (54.6)	50.0 (14.9-66.7)	0.459	<.001	0.285	<.001
Of the health care providers you have seen, how many made you feel belittled?	1.77 (3.10)	26.6 (31.7)	20.0 (0.0-40.0)	0.527	<.001	0.415	<.001
Of the health care providers you have seen, how many seemed to not listen to your concerns related to your symptoms (ie, how the symptoms affect the quality of your life or the ability to do certain aspects of your life such as work, exercise, have sex, etc)?	2.45 (3.32)	40.5 (40.4)	33.3 (0.0-61.9)	0.459	<.001	0.386	<.001
Of the health care providers you have seen, how many made you feel more isolated or alone at the end of a visit than before the visit?	2.36 (3.44)	36.8 (34.5)	33.3 (0.0-60.0)	0.565	<.001	0.418	<.001
Of the health care providers you have seen, how many made you feel your symptoms were “all in your head”?	1.47 (2.39)	22.8 (31.0)	0.0 (0.0-40.0)	0.512	<.001	0.406	<.001
Of the health care providers you have seen, how many made you feel that they did not believe your symptoms?	1.35 (2.32)	20.5 (30.9)	0.0 (0.0-33.3)	0.511	<.001	0.365	<.001
How many times did you change health care providers in the past because you felt dismissed or invalidated?	2.34 (3.41)	NA	NA	0.561	<.001	0.393	<.001

Abbreviation: NA, not applicable.

^a^ Calculated by using the number of clinicians exhibiting behavior reported by the patient, divided by the total number of past clinicians reported by each patient, then averaged across all patients.

**Table 3.** Patient Experience Questions and Reported Numerical Distress Ratings

Patient experience question	Patients answering yes, No. (%)	Patient-rated magnitude of experience-related distress (0-10 numeric rating scale), mean (SD)	Numerical distress rating, median (IQR)
Have you ever felt that a provider thought you were “crazy”?	176 (39.4)	7.39 (3.06)	8.0 (6.0-10.0)
Have you ever been told that you just need to relax more?	186 (41.6)	7.04 (3.21)	8.0 (5.0-10.0)
Have you ever experienced a medical encounter that caused you to feel unsafe in any way?	72 (16.8)	6.73 (3.84)	8.0 (1.0-10.0)
Have you ever been told that your symptoms were caused by your high levels of anxiety?	127 (28.4)	6.72 (3.46)	8.0 (4.3-10.0)
Have you ever seen a health care provider that recommended that you see a psychiatrist or psychologist instead of pursuing medical treatment for your symptoms?	92 (20.6)	6.56 (3.59)	8.0 (3.3-10.0)
I have been told the pain I experienced during my physical exam was normal despite reporting significant pain during the exam.	119 (26.6)	6.53 (3.43)	8.0 (4.0-10.0)
I was told my physical exam findings (anatomy and appearance of my vulva) were normal despite reporting significant pain during the exam.	236 (52.8)	6.52 (3.52)	8.0 (4.8-10.0)
Have you ever been told that you just “need a glass of wine” or a similar suggestion?	92 (20.6)	6.27 (3.72)	8.0 (1.0-10.0)
Have you ever been told that you are “too high strung” or “too uptight”?	104 (23.3)	6.71 (3.46)	7.0 (3.5-10.0)
Have you ever been told that you “must have been abused”?	34 (7.6)	5.17 (4.25)	5.0 (1.0-10.0)
Have you ever been told that your vaginal opening was just “too small”?	66 (14.8)	4.96 (3.75)	5.0 (1.0-8.8)

Participants reporting a higher percentage of supportive past practitioner behaviors on the survey also reported significantly lower overall distress ratings and lower rates of considering giving up on care (Table 2). Conversely, a higher percentage of past practitioners engaging in negative behaviors was associated with higher overall participant numerical distress rating and higher rate of considering giving up (Table 2). Older age was negatively associated with the number of past clinicians (Spearman ρ = −0.235; *P* < .001). There was also a negative association between higher age and all reported negative clinician behavior, including the proportion of past clinicians who made the patient feel crazy (Spearman ρ = −0.192; *P* < .001), made the patient feel isolated (Spearman ρ = −0.193; *P* < .001), or made the patient feel they did not believe their symptoms (Spearman ρ = −0.197; *P* < .001).

Patients provided a total of 1150 quotations to detail their survey responses. Of these, 122 were eliminated (85 relayed history without subjective detail, 26 were not applicable or similar, and 11 pertained to nongynecologic specialties). Overarching themes and subcategories that were extracted from the quotations, along with the number of applicable quotations, can be seen in Table 4. The most common themes patients reported were related to barriers to their care. Among these quotations, patient emphasis was often on frustration with clinician lack of knowledge (247 quotations), which patients often related to a lack of education or training in areas of medicine related to pain and sexual health. Frustration with the need to see an extensive list of clinicians (often associated with little improvement or fatigue due to so many visits) was also common (141 quotations).

**Table 4.** Thematic Analysis of Patient Quotations

Subcategory	Quotations, No.	Representative quotations
Barriers to care		
Clinician lack of knowledge	247	“I’m shocked at how it just seems like they stop at the ‘simple’ answer. It’s yeast or UTI, and it feels kind of dismissive after that. Something else has to be wrong—this is certainly not normal. I was last told ‘Your body wants to heal itself—just go let it.’”
		“It’s just so crazy to me how every medical professional I’ve seen has had such little knowledge on sexual dysfunction. They have just sent me around from doctor to doctor hoping somebody will figure it out.”
Frustration with multiple visits or clinicians	141	“I won’t give up because I need to get better, but after seeing so many providers that have been unable to help there have been times where I have been worn down and just didn’t know where to look next.”
System and support failure	86	“I would not wish this journey on even my worst enemy. It has felt hopeless for so many years and I dread each new interaction because my history reads like a novel. Friends ask me why I go to such expensive doctors and I have to tell them because the ones who take my insurance think I’m crazy.”
		“I was told that I had lichen with no explanation whatsoever of what it was only put this ointment on 14 d then 10 d and then twice a week then stop it may come back then you do the same all over again. I think it was very wrong to send me home like that and finding out on the internet everything about this.”
Difficulty with access to visits	51	“I walked away from pursuing this because I didn’t have the energy to deal with looking for new doctors, who can take that much time off to keep shopping around? Also, my insurance isn’t going to pay for it and I didn’t have the money in my younger years.”
		“During the first year of the onset of my symptoms I saw a gynecologist who told me my symptoms were only due to the overuse of clobetesol then refused to follow up once I was no longer using that medication.”
Expense (work, time, money)	30	“I feel like if you have the money to afford specialists who don’t take insurance, you can be helped. The more money, the better.”
Treatment difference based on gender of clinician or patient	24	“When I asked one of the male providers (after dealing with this for so many years)…he yelled at me and said that I’m fine and that testing my hormones would be a waste of health care dollars. Since then, I will never see another male provider.”
		“....If this were a male issue, I believe there would be multiple practices offering medical care. It is sad and scary that there is almost no place to turn for female pelvic pain matters despite living near major metropolitan areas.”
Concerning clinician behavior		
Clinician not listening or being dismissive	211	“I think they were probably listening but were just quick to give antibiotics or diflucan without trying to get to the bottom of why this keeps occurring.”
Clinician focus on mental health rather than physical symptoms or biomedical causes	120	“I not only have felt providers thought I was crazy, but now I believe that I am crazy. This doesn’t make any sense anymore and I feel as though perhaps they are correct about it being in my head.”
		“I believe the second you tell a doctor you have anxiety your quality of care immediately drops.”
Clinician poor bedside manner	105	“Most of the time, I felt they were rushed. I’ve heard everything from pelvic floor disorder, menopause, it’s normal, use hydrocortisone. Even when I was diagnosed with VIN III, a medical doctor said ‘no big deal. It hardly ever turns to cancer.’”
Clinician inflicting pain or assault during examination	86	“I had a biopsy done where they numbed me, but when they started taking the biopsy I could still feel everything. I told them and jolted a little bit, but he said they were almost done and continued.”
Lack of clinical investigation	82	“I have had providers simply refuse to do tests on me because they’ve said I’m ‘too young’ to worry about reproductive issues or that my pain is ‘normal for young women’ or that my pain ‘is probably just stress.’”
Clinician disregard for patient goals	58	“Providers who say things like ‘Intercourse is overrated anyway’ negate the fact that it’s important to me and to my partner. Providers that say ‘It’s just part of aging’ don’t address why it’s not ‘part of aging’ for my peers and even friends who are significantly older.”
Inappropriate plan (eg, sex or alcohol)	58	“...After this advice, I tried increasing amounts of alcohol to see if it would reduce my pain, but it did not. That was a very bad time.”
		“One doctor told me that it’s probably all in your head and just to increase sexual activity with your husband and it will get better.”
Patient confusion because of poor intraclinician consistency	34	“But also distressing to realize that Drs have drastically different ideas about what is normal. One Dr was in shock and I could see it on her face. Another one said, ‘There’s DEFINITELY inflammation.’ Another one said ‘This looks normal, maybe you have vulvadynia. I would try going to this functional nutritionist (without a degree).’ Another one said, ‘You have a minute clitoris and it looks like you’re losing some structure.’ No one said anything about the way that these observations were relevant to me practically or clinically.”
Clinician emphasis on normal examination	27	“Being told that they don’t see anything or there is nothing there, despite that I was in severe pain and crying and had been in that provider’s office almost every day for a week for these burning symptoms.”
Clinician disregard of regard for severity of symptoms	18	“Again majority of the physicians do not understand the impact this has on my life. I am virtually rendered worthless and dysfunctional. I cannot do the things I used to do. I fear doing the things I used to do.”
Emotional harms		
Patient emotional distress	193	“I did give up, and it’s cost me my whole adult life. Very little intimacy, so no boyfriends, no husband, no kids. I have a comfortable life but it’s not the one I wanted and it’s very lonely.”
Patient sense of futility	143	“This pain caused me to feel isolated and hopeless for many years, so each appointment where I was told there was nothing wrong added to the hopelessness.”
Patient self-doubt	45	“I feel like this is compounded by the fact that I tend to try not to be a complainer. As a patient you don’t want anything to be wrong, so it is easy to accept a non-diagnosis even when you know things are not right.”
Patient fear and worry	35	“I left worried that my clitoris might adhere to my clitoral hood...I’ve worried that I’m going to go bankrupt. I’ve laid awake at night in excruciating pain. I’ve been told to lose weight and go to therapy. Again and again. Until I became a specialist in motivational interviewing and behavioral change. I feel like I lost almost as much of my life to medical gaslighting as I did to physical pain.”
Patient lack of trust	31	“Feeling like there hasn’t been one that I trusted understood all of the issues going on so I haven’t been confident in treatment.”
Patient embarrassment	24	“I felt ashamed once at 20 when I had a nurse say ‘You have scarring on your vagina’ I felt ashamed like I had done something wrong and I do not [t]hink she was trying to shame me.”
Positive themes		
Clinician positive effort	164	“There have been two doctors who have listened to my multiple symptoms and have been very thoughtful around the solution, noting that it likely requires a multi-prong approach.”
Patient self-advocacy	89	“Many doctors said there was nothing wrong with me but I knew there was. I did my research and figured it was lichens….”
		“...I am my own advocate I live in this body I know I feel pain.”

Abbreviations: UTI, urinary tract infection; VIN, vulvar intraepithelial neoplasia.

Among patient concerns about behavior, the most common theme was clinicians who were dismissive or did not listen to the patient (211 quotations), with associated themes of ignoring physical symptoms in favor of mental health discussions (120 quotations) and generally poor bedside manner, which included quotations about rushed visits and lack of empathy (105 quotations). Patients relayed substantial emotional harms they experienced related to these behaviors, which included prominent themes of general emotional distress (193 quotations) and sense of futility (143 quotations). Although most of the quotations described negative interactions and harms, there were some less negative and positive themes, including 164 mentions of clinician effort or excellence and 89 mentions of patient self-advocacy.

## Discussion

In this cross-sectional study, patients presenting to a referral clinic for vulvovaginal disorders reported frequent experiences of gaslighting behaviors and other types of testimonial injustice. Patients who saw more past clinicians for their concern had higher levels of overall distress and more frequent gaslighting experiences. It follows that if a patient experiences inappropriate clinician behavior, they are more likely to seek a different clinician. In our referral population, challenging diagnoses and lack of widespread knowledge about sexual medicine may also cause patients to seek alternate care, increasing the risk of exposure to a clinician who engages in gaslighting or other harmful behavior.

Many patients (52.8%) with vulvovaginal disorders considered stopping care altogether because of their experiences; thus, the responses we received are likely an underrepresentation of this phenomenon, as patients who ceased further care are not represented. The experiences of patients with vulvovaginal disorders are unfortunately not unique in reproductive health, or in conditions that disproportionately affect women, transgender patients, and nonbinary patients. In obstetrics, gaslighting has been characterized as a form of violence that traumatizes birthing parents and has lasting impacts on their feelings about health care and their own bodies.^8^ In a small qualitative study of transgender and nonbinary patients seeking care for endometriosis, gaslighting behavior has been noted as a deterrent to seeking medical care.^16^ Our findings echo these and other studies that indicate that dismissive behavior by clinicians represent a barrier to patient care.

Harms of such behavior are not limited to psychological trauma and hesitancy about seeking care, although these are substantial.^12^ Patients in groups more likely to experience gaslighting are vulnerable to delays in cardiac and oncologic diagnoses, which have critical impacts on morbidity and mortality.^15,28,29,30^ In the vulvovaginal disease realm, delays can also cause substantial morbidity. Patients with dermatologic or neurologic conditions causing pain can develop worsening secondary pelvic floor muscle dysfunction, which can compound pain and anxiety, add bowel and bladder dysfunction, and lengthen time to recovery of function.^31,32^ Certain conditions such as persistent genital arousal disorder (PGAD) are associated with very high rates of distress and suicidal ideation (as high as 54%), so delay in seeking treatment can have profound effects on patient health and safety.^33,34^

Direction of effect or causation cannot be determined from this study, but it is notable that overall distress and consideration of giving up care were correlated with reports of adverse clinician experiences. Certainly, some patients with more severe disease or underlying illness may experience more distress from harmful interactions, but it is also possible that their experience of illness is worsened by the interactions they have with clinicians. Unlike medication adherence, disease severity, or treatment adverse effects, behavior during appointments is the element of care that is most under control of the clinician. Simply limiting harmful behaviors such as recommending alcohol consumption or sexual activity in the context of vulvovaginal pain may be associated with lower patient distress, and being helpful—even when lacking knowledge—can substantially improve patient experience. Indeed, 1 study examining health care experiences in people with PGAD found that the more helpful a practitioner was perceived to be, the lower the participants’ PGAD or genitopelvic dysesthesia symptom severity score.^35^ Educational intervention in trainees and large clinician groups are a natural next step to limiting such harm; interventions could be studied by preintervention and postintervention surveys of clinicians and patients.

The development of metrics to measure experiences related to epistemic injustice, or gaslighting in particular, has been limited. Most studies of such behaviors are qualitative in nature, but some instruments have been developed to measure gaslighting in the context of personal relationships. The first such instrument, the Gaslighting Questionnaire, was described in a popular book in 2007 (reissued in 2018)^36^ and was directed generally toward interpersonal relationships, but has subsequently been evaluated in 1 peer-reviewed publication.^37^ Several more recent statistically validated instruments have been developed on the basis of literature reviews or prior instruments to measure intimate relationship experiences with gaslighting.^38,39,40^ One of these scales derived items from patient experiences obtained through a focus group of participants affected by domestic abuse, with subsequent review of items by subject-matter experts.^41^ To the best of our knowledge, the current study represents the first publication of a patient-centered metric of behaviors like gaslighting for a medical context.

The dearth of validated instruments for the measurement of medical gaslighting and injustice limits our ability to measure the success of interventions to improve patient care; measurement of the success of such interventions depends on reliable metrics. Further development of such instruments and validation in more-diverse clinical contexts are in keeping with prior calls for clinicians to address gaslighting behavior and other forms of epistemic injustice given their substantial impact on patient care.^5^ Incorporation of teaching reproductive justice principles has been proposed as an antidote to gaslighting in the context of social work, and certainly this is a component of improvement in gynecology care as well.^3^

### Strengths and Limitations

This study used a collaborative approach to ensure that patient concerns regarding gaslighting and other dismissive behavior were at the forefront of a novel survey measuring such harms. Patient testimonies were used to originate instrument items, and feedback from national advocacy organizations was used to tailor responses to be patient centered. The survey of patients in our practice had a very high completion rate and a large sample size relative to other similar studies, lending further credence to the findings. Numerical scales characterized patient distress regarding clinician gaslighting behaviors beyond simple binary responses. The study also allowed patients to provide narrative responses to further enrich our understanding of their quantitative responses. These qualitative responses were analyzed in detail by the study team to further characterize patient experiences.

The study also has limitations that should be mentioned. The survey instrument has not been statistically validated. There is also most certainly recall bias as patients were discussing experiences from variable time durations and frequency in the past. Selection bias is also a concern, as the patients presenting the clinic are often referred because of their more challenging conditions and history of many evaluations before presentation without resolution of symptoms.

Our study was also limited to a single clinic population, which may make results difficult to generalize to more diverse socioeconomic and racial and ethnic populations; more vulnerable populations experience gaslighting at a higher rate.^14,16,28^ Our clinic population is not diverse, and the cost of clinic visits also represents a potential barrier to care. A large proportion of patients in our sample also considered stopping care; thus, a substantial number of patients who actually stopped care are not represented in our sample. Consequently, the true instance of harmful clinician behaviors may be higher than our results suggest. However, given the referral population, it is also possible that our patient population has been disproportionately highly subjected to these behaviors and that our survey data overstate the problem.

## Conclusions

In this cross-sectional study of patients with vulvovaginal disorders, participants frequently reported clinician behavior that was dismissive, invalidating, and led them to question their own experience or consider cessation of care altogether. The study was framed in terms quantifying the harm conferred by gaslighting and other dismissive clinician behavior; future work must be directed toward determining best practices for clinicians and interventions to reduce such harm. Subsequent study should also be directed toward validating an instrument to quantify care experiences in a more-diverse patient population and outside of a referral clinic.

## Notes

Supplement 1. **eAppendix.** Gaslighting and Sexual Medicine (GASM) Instrument

Supplement 2.
Data Sharing Statement
