# Supplementary material for: Experiences of Care and Gaslighting in Patients With Vulvovaginal Disorders
Source: JAMA Netw Open. 2025 May 8;8(5):e259486. doi: 10.1001/jamanetworkopen.2025.9486 (PMC12062909; doi:10.1001/jamanetworkopen.2025.9486)
Supplement: Supplement 1. — eAppendix. Gaslighting and Sexual Medicine (GASM) Instrument [file jamanetwopen-e259486-s001.pdf]

# Supplemental Online Content

Moss CF, Chinna-Meyyappan A, Skovronsky G, et al. Experiences of care and gaslighting in patients with vulvovaginal disorders. *JAMA Netw Open*. 2025;8(5):e259486. doi:10.1001/jamanetworkopen.2025.9486

**eAppendix.** Gaslighting and Sexual Medicine (GASM) Instrument

This supplemental material has been provided by the authors to give readers additional information about their work.

© 2025 Moss CF et al. *JAMA Network Open*.

**eAppendix. Gaslighting and Sexual Medicine (GASM) Instrument**

1. How many medically trained health care providers (doctors, midwives, nurse practitioners, or physician assistants) have you seen to address your pelvic/vulvar pain in the past?

   A)1 B)2 C)3 D)4 E)5 F)6 G)7 H)8 I)9 J)10 K) greater than 10
   If greater than 10, how many? (open text field)

   Do you have any comments about the number of healthcare providers you have seen? (open text field)

2. Of the health care providers you have seen, how many have partnered with you in making the plan for your care?

   A)1 B)2 C)3 D)4 E)5 F)6 G)7 H)8 I)9 J)10 K) greater than 10
   If greater than 10, how many? (open text field)

   Please feel free to share any additional information that you are willing to share, without revealing your name or the name/s of any health care providers. (open text field)

3. Of the health care providers you have seen, how many made you feel supported?

   A)1 B)2 C)3 D)4 E)5 F)6 G)7 H)8 I)9 J)10 K) greater than 10
   If greater than 10, how many? (open text field)

   Please feel free to share any additional information that you are willing to share, without revealing your name or the name/s of any health care providers. (open text field)

4. Of the healthcare providers you have seen, how many made you feel like they were not really listening to your symptoms?

   A)1 B)2 C)3 D)4 E)5 F)6 G)7 H)8 I)9 J)10 K) greater than 10
   If greater than 10, how many? (open text field)

   Please feel free to share any additional information that you are willing to share, without revealing your name or the name/s of any health care providers. (open text field)

5. Of the health care providers you have seen, how many made you feel belittled?

   A)1 B)2 C)3 D)4 E)5 F)6 G)7 H)8 I)9 J)10 K) greater than 10
   If greater than 10, how many? (open text field)

   Please feel free to share any additional information that you are willing to share, without revealing your name or the name/s of any health care providers(open text field)

© 2025 Moss CF et al. *JAMA Network Open*.

6. Of the health care providers you have seen, how many seemed to not listen to your concerns related to your symptoms (i.e. how the symptoms affect the quality of your life or the ability to do certain aspects of your life such as work, exercise, have sex, etc) ?

   A)1 B)2 C)3 D)4 E)5 F)6 G)7 H)8 I)9 J)10 K) greater than 10
   If greater than 10, how many? (open text field)

   Please feel free to share any additional information that you are willing to share, without revealing your name or the name/s of any health care providers. (open text field)

7. Of the healthcare providers you have seen, how many made you feel more isolated or alone at the end of a visit than before the visit?

   A)1 B)2 C)3 D)4 E)5 F)6 G)7 H)8 I)9 J)10 K) greater than 10
   If greater than 10, how many? (open text field)

   Please feel free to share any additional information that you are willing to share, without revealing your name or the name/s of any health care providers. (open text field)

8. Of the health care providers you have seen, how many made you feel your symptoms were "all in your head?"

   A)1 B)2 C)3 D)4 E)5 F)6 G)7 H)8 I)9 J)10 K) greater than 10
   If greater than 10, how many? (open text field)

   Please feel free to share any additional information that you are willing to share, without revealing your name or the name/s of any health care providers. (open text field)

9. Of the healthcare providers you have seen, how many made you feel that they did not believe your symptoms?

   A)1 B)2 C)3 D)4 E)5 F)6 G)7 H)8 I)9 J)10 K) greater than 10
   If greater than 10, how many? (open text field)

   Please feel free to share any additional information that you are willing to share, without revealing your name or the name/s of any health care providers. (open text field)

10. How many times did you change healthcare providers in the past because you felt dismissed or invalidated?

    A)1 B)2 C)3 D)4 E)5 F)6 G)7 H)8 I)9 J)10 K) greater than 10
    If greater than 10, how many? (open text field)

© 2025 Moss CF et al. *JAMA Network Open*.

Please feel free to share any additional information that you are willing to share, without revealing your name or the name/s of any health care providers. (open text field)

11. Overall, how distressing would you rate your encounters with medically trained healthcare providers in relation to your pelvic/vulvar pain?

    0 (not at all distressing) to 10 (extremely distressing)

12. I was told my physical exam findings (anatomy and appearance of my vulva) were normal despite reporting significant pain during the exam.

    Yes/No

13. If yes, how distressing was this experience for you?

    0 (not at all distressing) to 10 (extremely distressing)

    Please feel free to share any additional information that you are willing to share, without revealing your name or the name/s of any health care providers. (open text field)

14. I have been told the pain I experienced during my physical exam was normal despite reporting significant pain during the exam

    Yes/No

15. If yes to the above question, how distressing was this experience for you?

    0 (not at all distressing) to 10 (extremely distressing)

    Please feel free to share any additional information that you are willing to share, without revealing your name or the name/s of any health care providers. (open text field)

16. Have you ever felt that a provider thought you were “crazy?” Yes/No

17. If yes to the above question, how distressing was this experience for you?

    0 (not at all distressing) to 10 (extremely distressing)

    Please feel free to share any additional information that you are willing to share, without revealing your name or the name/s of any health care providers. (open text field)

18. Have you seen a healthcare provider that recommended that you see a psychiatrist or psychologist instead of pursuing medical treatment for your symptoms?

19. If yes to the above question, how distressing was this experience for you?

© 2025 Moss CF et al. *JAMA Network Open*.

0 (not at all distressing) to 10 (extremely distressing)

Please feel free to share any additional information that you are willing to share, without revealing your name or the name/s of any health care providers. (open text field)

20. Have you ever been told that you just need to relax more? Yes/No

21. If yes to the above question, how distressing was this experience for you?

    0 (not at all distressing) to 10 (extremely distressing)

    Please feel free to share any additional information that you are willing to share, without revealing your name or the name/s of any health care providers(open text field)

22. Have you ever been told that you are “too high strung” or “too uptight”? Yes/No

23. If yes to the above question, how distressing was this experience for you?

    0 (not at all distressing) to 10 (extremely distressing)

    Please feel free to share any additional information that you are willing to share, without revealing your name or the name/s of any health care providers. (open text field)

24. Have you ever been told that you just “need a glass of wine?” or a similar suggestion? Yes/No

25. If yes to the above question, how distressing was this experience for you?

    0 (not at all distressing) to 10 (extremely distressing)

    Please feel free to share any additional information that you are willing to share, without revealing your name or the name/s of any health care providers. (open text field)

26. Have you been told that your symptoms were caused by your high levels of anxiety? Yes/No

27. If yes to the above question, how distressing was this experience for you?

    0 (not at all distressing) to 10 (extremely distressing)

    Please feel free to share any additional information that you are willing to share, without revealing your name or the name/s of any health care providers. (open text field)

28. Have you ever been told that you “must have been abused?” Yes/No

29. If yes to the above question, how distressing was this experience for you?

© 2025 Moss CF et al. *JAMA Network Open*.

0 (not at all distressing) to 10 (extremely distressing) Respondent skipped this question

Please feel free to share any additional information that you are willing to share, without revealing your name or the name/s of any health care providers. (open text field)

30. Have you ever been told that your vaginal opening was just “too small?” Yes/No
31. If yes to the above question, how distressing was this experience for you?

    0 (not at all distressing) to 10 (extremely distressing)

    Please feel free to share any additional information that you are willing to share, without revealing your name or the name/s of any health care providers. (open text field)

32. Have you ever experienced a medical encounter that caused you to feel unsafe in any way? Yes/No
33. If yes to the above question, how distressing was this experience for you?

    0 (not at all distressing) to 10 (extremely distressing) 10

    Please feel free to share any additional information that you are willing to share, without revealing your name or the name/s of any health care providers. (open text field)

34. Have you ever considered giving up seeking additional care because you felt that your concerns were not addressed? Yes/No

    Please feel free to share any additional information that you are willing to share, without revealing your name or the name/s of any health care providers. (open text field)

35. Have you ever considered giving up seeking additional care because you were made to feel there weren’t any other providers who could help you? Yes/No

    Please feel free to share any additional information that you are willing to share, without revealing your name or the name/s of any health care providers. (open text field)

36. Are you African-American/ Black, Alaskan Native, American Indian, Asian, White, Native Hawaiian or other Pacific islander, or some other race? Please choose all that apply
37. Are you of Spanish, Hispanic or Latino origin or descent? Please choose all that apply

© 2025 Moss CF et al. *JAMA Network Open*.
