# Supplementary material for: Experiences of Care and Gaslighting in Patients With Vulvovaginal Disorders
Source: JAMA Netw Open. 2025 May 8;8(5):e259486. doi: 10.1001/jamanetworkopen.2025.9486 (PMC12062909; doi:10.1001/jamanetworkopen.2025.9486)
Supplement: Supplement 2. — Data Sharing Statement [file jamanetwopen-e259486-s002.pdf]

Data Sharing Statement

Moss. Experiences of Care and Gaslighting in Patients With Vulvovaginal Disorders. *JAMA Netw Open*. Published May 08, 2025. doi:10.1001/jamanetworkopen.2025.9486

Data
Data available: No

Additional Information
Explanation for why data not available: Patients were asked to not included identifiable data, but the sensitive narrative responses they submitted were often detailed and potentially could identify past clinicians or personal details.
